# Ageing, Health and Equity—Broad Perspectives Are Needed to Understand and Tackle Health Challenges of Ageing Societies

**DOI:** 10.3390/ijerph15030457

**Published:** 2018-03-06

**Authors:** Hajo Zeeb, Heinz Rothgang, Ingrid Darmann-Finck

**Affiliations:** 1 Leibniz Institute for Prevention Research and Epidemiology—BIPS, 28359 Bremen, Germany; 2 Health Sciences Bremen, University of Bremen, 28359 Bremen, Germany; rothgang@uni-bremen.de (H.R.); darmann@uni-bremen.de (I.D.-F.); 3 SOCIUM—Research Center on Inequality and Social Policy, University of Bremen, 28359 Bremen, Germany; 4 Institute for Public Health and Nursing Research (IPP), University of Bremen, 28359 Bremen, Germany

**Keywords:** ageing, health, equity, public health

Demographic change and the evolving demands on healthcare systems, especially in the provision of healthcare and long-term care for a growing number of older people, are among the greatest social challenges of the next decades. This is particularly true for the WHO European region that includes 9 of the 10 countries with the longest life expectancy globally. The pursuit of health equity in ageing societies raises several questions: On the one hand, there is persisting health inequities that are related to a social gradient in health, in and between countries. On the other hand, as resources are scarce, social challenges to healthcare and the healthcare system will always entail questions of distributive justice. This is even more important as the fair and effective functioning of health care and social systems is of particular relevance to older people, who carry a much higher overall burden of disability than, for example, middle-aged population groups. Most of the health problems of older age are related to chronic diseases, and this underlines the role of prevention and health promotion throughout the life course to help reduce this burden due to non-communicable diseases [1]. However, the feasibility and effectiveness of health promotion interventions for seniors often remain unclear. New avenues of intervention delivery, e.g., through ehealth approaches are an active area of research at present (e.g., [2]), and overall the topic of ageing and health has become a major Public Health research field over recent years.

At the University of Bremen, interdisciplinary Public health and Nursing researchers working in the high profile research area Health Sciences organized a two-day conference in June 2017 to discuss issues of ageing, health, and equity. The conference covered preventive as well as health service and long-term care aspects, and also discussed training and qualification of healthcare professionals. We were interested to understand more deeply health problems of the elderly as well as potential solutions, and in particular which societal and social system aspects or interventions contribute to or alleviate inequalities and/or strengthen equity. While there was a clear empirical focus, new insights into theories, concepts, and research methods were welcome. Conference-related as well as additional manuscripts were then submitted and reviewed for this special issue of the IJERPH. The special issue includes three focus areas: (i) methods-oriented approaches, (ii) social inequality and health among the elderly and inequality in the utilization of long-term care, and (iii) the health status of and service provision for people in need of long-term care.

## 1. Methods-Oriented Approaches

A methods-oriented approach to specific health and healthcare topics among the elderly is presented by papers from Dorant and Krieger [3], Schilling and Gerhardus [4], and Bunt et al. [5].

Dorant and Krieger [3] conducted participatory health research to explore and improve a family caregiver support program, with care professionals acting as co-researchers. Long-term care is predominantly provided in the home setting, with strong involvement of family members. The family caregiver support system is aptly considered as a complex intervention in the geriatric setting, and the many professions that are involved in geriatric care are considered as (still learning) experts also for research development and conduct. The authors provide first insight into processes of research co-creation in the geriatric setting, with a focus on the involvement of service providers. As further step in participatory health research, all of the stakeholders, including actual family caregivers, and eventually service recipients, might be included, but this needs other careful preparatory steps.

This is the topic of Schilling and Gerhardus’ [4] work who start with the premise that “little is known about how to involve older people” in health research. In their review of methods for involvement of older people in health research they finally included 9 studies from the UK, all but one focusing on people with dementia. Overall, the authors conclude that research involvement of seniors is feasible, but very careful consideration has to be given to issues, such as the research setting, appropriate communication, and limits in mobility, as well as self-confidence and trust among the elderly with age-related health problems.

Some of these restraints are also covered by the concept of social frailty, which describes the different stages of loss of social resources, abilities, and activities that are relevant to fulfill basic social needs. Research needs proper instruments, and Bunt et al. [5] provide detailed insight into the cross-cultural adaptation of a specific tool, the Social Vulnerability Index, for use in the Dutch context. Through several Delphi rounds, the translated instrument was adapted and then tested in a small sample of older adults. While its use was generally shown to be feasible, marked heterogeneity in the application time between two experts using the instrument highlights one of the remaining challenges, and more experience with measuring and understanding social frailty is clearly needed. This includes a full assessment of psychometric properties of the index.

## 2. Social Inequality and Health among the Elderly and Inequality in the Utilization of Long-Term Care

The papers of Nazroo [6], Hoebel et al. [7], Schönbach et al. [8], and Schoenmakers et al. [9] focus on social inequality and health among the elderly, alternatively investigated under a class or ethnicity-cultural lens.

James Nazroo [6] uses data from the English Longitudinal Study of Ageing (ELSA) to carefully analyze inequality in the post-retirement period. He puts a focus on healthy life expectancy and its variation across levels of socioeconomic position. A beauty of the available data from ELSA lies in the diversity of measures both regarding socioeconomic inequality and health and wellbeing. Nazroo then draws on Bourdieu for a theory-grounded (and life-phase adequate) assessment of causal mechanisms relating class with health and wellbeing in later life. He notes high risks of moving into social detachment among the least affluent, which in turn is related to markedly poorer health.

For Germany, Hoebel et al. [7] analyse inequalities and perceived unmet needs among participants of the German Health Update study aged 50–85 years. Their analysis indicates that the socioeconomic differences in health remain present across the age-range, but appear to narrow at older ages and more so among men then among women for several of the self-rated aspects considered. The different potential explanations put forward, including selective survival and the age-as-a-leveler hypothesis, make an interesting read and stimulate critical discussion.

Schönbach et al. [8] provide deeper insight into an example of health-related behavior and show that physical activity as a key factor of healthy aging, measured as sports participation, takes a positive leap at entry to retirement, but social differentials remain. A migrant background as well as the level of acculturation only had minor effects on the enhanced post-retirement physical activity levels in this analysis of German Socio-Economic Panel data.

One import factor for prevention as well as physical and mental health care is the availability of social networks. What exactly this role might be with respect to access to psychosocial care for migrant populations in the Netherlands is the topic of Schoenmakers and coauthors’ work [8]. Their qualitative study highlights the role of children in navigating towards psychosocial care. It also shows that social networks of elderly migrants are generally not sufficient to adequately support psychosocial needs, and sometimes contribute to stresses and worries. Enabling factors include, good language proficiency and good mental health literacy of individuals and their networks, among others.

## 3. Health Status of and Service Provision for People in Need of Long-Term Care

Inequality in the utilization of long-term care and the health status of residents of nursing homes are analyzed by Jacobs et al. [10], Ilinca et al. [11], and Frisina Doetter and Schmid [12].

Physical frailty in old age results in hip fractures with disproportionate frequency as age increases. Jacobs et al. [10] investigate hip fracture frequency and determinants among nursing home residents and confirm a markedly increased risk during the immediate time period after first admission to the home. Incidence rates for women were 20% higher than for men. It seems hard to conceive active preventive measures for this problem, but clearly a heightened awareness of nursing home staff during the first months of new residents is warranted.

Long-term care arrangements become more and more important in European countries. Ilinca et al. [11] analyze inequality and inequity among older European populations with respect to home care. SHARE data from 2013 are analyzed using the Concentration Index, a measure of health inequality related to socio-economic status, and further decomposition of explanatory factors. The authors can show that use of long-term care differ between income groups within countries even after controlling for care need, with poorer households using more long-term home care. They highlight the importance of clear definitions with respect to need factors as differences in definition—as shown in the paper—lead to contradictory results.

If the need of long-term care is so high that families can no longer cope with it, traditionally, nursing homes have been the only alternative. When the mandatory long-term care insurance was founded in Germany in 1994, its major benefits were cash payments to (partly) compensate family care-givers, in kind benefits that might support family care-givers and benefits for nursing home care. If home care is not or no longer possible, nursing home care once again seems to be the natural alternative. In the last decade, however, the interest in innovative care models that provide more choice and flexibility to beneficiaries has increased. Frisina Doetter, and Schmid [12] examine one kind of alternative, i.e., ‘shared housing’, where a small group of people rent private rooms, while sharing a common space, domestic support, and nursing care. Based on interviews and secondary data, they analyze in how far such new forms of care provision may provide an alternative to nursing home care for a larger population of beneficiaries than presently seen in Germany, thus increasing equity.

## 4. Synthesis, Conclusions, Research Needs and Opportunities

The contributions to this special issue have commonalities in their consideration – at different levels—of socioeconomic factors shaping health in advanced age. In some cases, life events including retirement or the need of long-term care or admission to a nursing home, are the cornerstone shaping subsequent health-related dynamics or occurrences, in other cases, the perspective centers on larger age groups and their comparative health and social status. These approaches alone provide for multiple different insights into the health and health needs of ageing populations and service provision to them. The special issue additionally gives exemplary guidance on many different data sources and research methods that can be used in this field, and the involvement of caregivers as well as the elderly themselves in research is likely to be both challenging and necessary for increasing relevance and transferability of research findings in day-to-day settings. Careful reading of the papers in this issue also brings to light two further messages: socioeconomic differentials in health relevant for older age are taking their course much earlier in life, but they continue to be dynamic. Also: ageing and retirement brings new opportunities, be it in terms of changing physical activity levels or of active social engagement, which may also include participation in Public Health research on ageing, health, and equity.
